# Rapid Progression of Blastic Plasmacytoid Dendritic Cell Neoplasm without Extracutaneous Manifestation

**DOI:** 10.4274/tjh.2013.0368

**Published:** 2015-02-15

**Authors:** Guohua Yu, Xin Huang, Yuqing Huo, Tingguo Zhang, Zifen Gao

**Affiliations:** 1 Shandong University Faculty of Medicine, Department of Pathology, Jinan, China; 2 Medical College of Qingdao University, Affiliated Yantai Yuhuangding Hospital, Clinic of Pathology, Yantai, China; 3 Peking University Faculty of Medical Science, Health Science Center, Department of Pathology, Beijing, China; 4 Affiliated Hospital, Binzhou Medical College, Department of Oncology, Binzhou, China

**Keywords:** Blastic plasmacytoid dendritic cell neoplasm, Cutaneous involvement

A 58-year-old Chinese male presented with a 3-month history of multiple purple nodules on the back of his left shoulder and on his back. The skin lesions initially appeared as maculopapules and grew progressively. Upon admission, the purplish nodules measured from 1 cm to 6 cm in diameter (Figure 1A). Blood results and image examination were normal. Tumor cells were not found in the examination of bone marrow aspiration and biopsy. With the patient’s approval, a skin biopsy was performed. On gross examination, the cut surface of the skin mass was tan-white with obscure boundaries and a firm consistency (Figure 1B). Histological examination demonstrated a dense and diffuse infiltrate of monomorphous medium-sized cells in the dermis and subcutis (Figure 1C). Immunohistochemistry showed that tumor cells were positive for CD4 (Figure 1D), CD56 (Figure 1E), CD123 (Figure 1F), and CD43 and were negative for CD3, CD5, CD20, CD30, granzyme B, and TdT. In situ hybridization testing for the Epstein-Barr virus was negative. The patient was diagnosed with blastic plasmacytoid dendritic cell neoplasm and received 4 courses of CHOP (cyclophosphamide + adriamycin + leurocristine + prednisone) chemotherapy. After the treatment, he suffered persistent high fever and pancytopenia and died 3 months later. Informed consent was obtained.

## Figures and Tables

**Figure 1:**
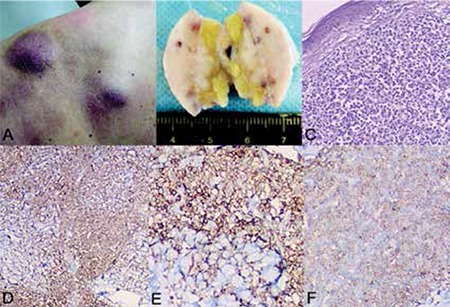
Macroscopic, microscopic, and immunophenotypic findings of the case: (A) Multiple purple skin nodules on the back of his left shoulder; (B) the ill-defined mass had a solid and tan-white cut surface and was firm in consistency with visible infiltration of subcutaneous tissue; (C) H&E staining shows diffuse infiltration of the tumor cells in the dermis and subcutis (4x); immunophenotypic examination revealed that the tumor cells were (D) CD4-positive, (E) CD56-positive, and (F) CD123-positive.

